# National Cleft and Craniofacial Awareness and Prevention Month

**Published:** 2013-07-05

Annually, approximately 7,000 U.S. infants are born with a cleft palate alone or a cleft lip with or without cleft palate (1). Other common craniofacial birth defects include craniosynostosis (when the skull sutures fuse prematurely) and microtia/anotia (when an infant’s ear is small and poorly formed or missing). To increase awareness about these conditions, July is designated as National Cleft and Craniofacial Awareness and Prevention Month.

CDC and its partners work to better understand causes of cleft and craniofacial defects and how these conditions affect children and their families by focusing on risk factors, health-care service use, access to care, quality of life, health outcomes, and management and treatment of these conditions. Research has identified risk factors for cleft lip with or without cleft palate, including maternal diabetes (2), smoking (3), and certain medications (4,5). For craniosynostosis, research has shown an increased risk associated with maternal thyroid disease or its treatment during pregnancy (6). Parameters of care recently were developed to help treat children with craniosynostosis (7).

Health-care providers should encourage patients who are thinking about becoming pregnant to control diagnosed diabetes and quit smoking, and should work with patients to make informed decisions about medication treatment during pregnancy. Information regarding National Cleft and Craniofacial Awareness and Prevention Month is available at http://www.nccapm.org/about.html. Additional information on craniofacial birth defects is available at http://www.cdc.gov/ncbddd/features/cleft-awareness-july2013.html.
